# Non-fatal suicidal behaviour in rural Ethiopia: a cross-sectional facility- and population-based study

**DOI:** 10.1186/s12888-016-0784-y

**Published:** 2016-03-22

**Authors:** Abebaw Fekadu, Girmay Medhin, Medhin Selamu, Tsion Shiferaw, Maji Hailemariam, Sujit D Rathod, Mark Jordans, Solomon Teferra, Crick Lund, Erica Breuer, Martin Prince, Tedla W. Giorgis, Atalay Alem, Charlotte Hanlon

**Affiliations:** Department of Psychiatry, School of Medicine, College of Health Sciences, Addis Ababa University, Addis Ababa, Ethiopia; Department of Psychological Medicine, Institute of Psychiatry, Psychology and Neuroscience, Centre for Affective Disorders, King’s College London, London, UK; Aklilu Lemma Institute of Pathobiology, Addis Ababa University, Addis Ababa, Ethiopia; Department of Population Health, London School of Hygiene and Tropical Medicine, London, UK; Department of Research and Development, HealthNet Transcultural Psychosocial Organisation, Amsterdam, The Netherlands; Department of Psychiatry and Mental Health, Alan J Flisher Centre for Public Mental Health, University of Cape Town, Cape Town, South Africa; Health Service and Population Research Department, Institute of Psychiatry, King’s College London, London, UK; Republic of Ethiopia Federal Ministry of Health, Addis Ababa, Ethiopia

**Keywords:** Suicide attempt, Suicidal ideation, Suicidal plan, Suicidal behaviour, Low and middle income country, Ethiopia

## Abstract

**Background:**

Injury related to self-harm is one of the leading causes of global disease burden. As a formative work for a programme to implement comprehensive mental healthcare in a rural district in Ethiopia, we determined the 12-month prevalence of non-fatal suicidal behaviour as well as factors associated with this behaviour to understand the potential burden of the behaviour in the district.

**Method:**

Population-based (*n* = 1485) and facility-based (*n* = 1014) cross-sectional surveys of adults, using standardised, interview-based measures for suicidality (items on suicide from the Composite International Diagnostic Interview), depressive symptoms (the Patient Health Questionnaire) and alcohol use disorders (Alcohol Use Disorder Investigation Test; AUDIT).

**Results:**

The overall 12-month prevalence of non-fatal suicidal behaviour, consisting of suicidal ideation, plan and attempt, was 7.9 % (95 % Confidence Interval (CI) = 6.8 % to 8.9 %). The prevalence was significantly higher in the facility sample (10.3 %) compared with the community sample (6.3 %). The 12-month prevalence of suicide attempt was 4.4 % (95 % CI = 3.6 % to 5.3 %), non-significantly higher among the facility sample (5.4 %) compared with the community sample (3.8 %). Over half of those with suicidal ideation (56.4 %) transitioned from suicidal ideation to suicide attempt. Younger age, harmful use of alcohol and higher depression scores were associated significantly with increased non-fatal suicidal behaviours. The only factor associated with transition from suicidal ideation to suicide attempt was high depression score. Only 10.5 % of the sample with suicidal ideation had received any treatment for their suicidal behaviour: 10.8 % of the community sample and 10.2 % of the facility sample. Although help seeking increased with progression from ideation to attempt, there was no statistically significant difference between the groups.

**Conclusion:**

Non-fatal suicidal behaviour is an important public health problem in this rural district. A more in-depth understanding of the context of the occurrence of the behaviour, improving access to care and targeting depression and alcohol use disorder are important next steps. The role of other psychosocial factors should also be explored to assist the provision of holistic care.

## Background

Injury related to “self-harm” is one of the leading causes of global disease burden in many parts of the world [1]. The burden is particularly high in high-income Asia-Pacific countries and Eastern Europe, where the burden of disease caused by self-harm is ranked fifth and sixth, respectively [1]. Self-harm related disease burden is reported to be relatively lower in Western Africa (ranked 69th) and Eastern sub-Saharan Africa (ranked 32nd). However, given the overall scarcity of data and stigma surrounding self-harming behaviours, this apparently lower burden in Eastern sub-Saharan Africa may be due to under-reporting or the dearth of studies reporting on the subject. For example, in a large scale international study coordinated by the World Health Organization involving 11 “developing” and 10 “developed” countries, the 12 month prevalence of suicidal ideation, plans and attempts were slightly higher among participants from developing countries [2]. In fact, 85 % of the over 800,000 annual suicide deaths [3] are likely to occur in low and middle income countries (LMICs), where resources to prevent suicide deaths are severely limited. Suicide might indeed be a hidden cause of death in traditional societies because of the high level of stigma and associated religious and cultural condemnation [4, 5]. For example, in a study of 100 key informants, primarily consisting of community and religious leaders from Ethiopia [4], people who commit suicide were considered “condemned sinners” who do not deserve appropriate funeral rituals but should be buried in isolated unmarked place. Those who attempt suicide were “feared” and considered “cruel” and “untrustworthy”.

Yet data from LMICs remain sparse and, where available, the incidence of suicide and non-fatal suicidal behaviour shows marked variability [6]. The potential role of rural residence on rate of suicidal behaviour is also not adequately explored. An old study evaluating the rate of suicide in West Africa [7] reported a higher rate of suicide among rural residents; however, the risk of under-reporting in this study is very high.

In studies from Ethiopia, the reported lifetime prevalence of suicide attempts varied between 0.9 and 14.8 % [8, 9]. The rate of suicide attempts was particularly high among the homeless in Addis Ababa [10] and those with mental disorders [11–13]. Studies from other African countries report a similarly variable prevalence of suicidal behaviour. In a study conducted in a primary care facility in Kenya, 12.7 % of participants were reported to be at risk of suicide, although the specific rates for suicide attempt or ideation were not provided [14]. In Morocco, the one month prevalence of suicidal ideation was 6.3 % [15]. A study from Nigeria reported a 3.2 % prevalence of suicidal ideation and 0.7 % for suicide attempt. [16] In South Africa, the lifetime prevalence of suicide ideation, plans and attempts were 9.1, 3.8 and 2.9 % respectively [17]. Despite the relatively large variation, these figures indicate that suicidal behaviour is an important problem in Africa. Several risk factors for suicidal behaviour have been reported at the health system, community and individual level. The system level risk factors focus on access to means of suicide and health care. The individual factors include demographic features (female gender, younger age, lower education level, unmarried status) while stigma and discrimination are reported to be relevant community factors [3]. Mental disorders are perhaps the most important risk factors. Mood disorders, such as depression and bipolar disorder, impulse control disorder, and substance abuse are the most important disorders [18]. Given the dearth of data, particularly among rural communities, and the inconsistencies in prevalence figures, further studies to establish the prevalence and potential risk factors of non-fatal suicidal behaviour, such as demographic factors, depressive symptoms and alcohol use disorder [18], are required.

Moreover, non-fatal suicidal behaviours share risk factors with completed suicide [6] and are strong predictors of suicide even many years after the attempt [19]. It is therefore essential to understand non-fatal suicidal behaviour in order to develop intervention strategies which would also contribute to the prevention of suicide. In this study, we determined the prevalence of non-fatal suicidal behaviour (i.e. suicidal ideation, suicidal plan and suicide attempt). As in other studies [20], we defined suicidal ideation as the engagement in thoughts about self-inflicted harm to end one’s life. Suicidal plan was defined as “the formulation of a specific method through which one intends to die”. Suicide attempt represented “engagement in potentially self-injurious behaviour in which there is at least some intent to die.”

The study was conducted as part of the formative phase of the Programme for Improving Mental Healthcare (PRIME) [21], a multi-country research programme consortium, which works with Ministries, local governments and communities to integrate mental health services in primary healthcare.

## Methods

The data reported here were collected from a cross-sectional population-based survey and a facility-based survey of adults aged 18 years and above.

### Setting

The study was conducted in the Sodo district, Gurage Zone, Southern Nations, Nationalities and Peoples Region (SNNPR); a predominantly rural district located about 100 km south of the capital city, Addis Ababa. The population of the district is 161,952 persons (79,356 men; 82,596 women) living in 58 sub-districts (*kebeles)* [22]. The largest ethnic group in the district is Sodo Gurage (85.3 %) followed by Oromo (11.6 %) and Amhara (1.5 %) and Amharic is the official language. The majority of the population are Orthodox Christian (~97.0 %) with Muslims making up ~2.3 %. Within Sodo district, primary care services comprise eight public health centres, staffed by nurses and health officers, and 58 health posts (community-based facilities) staffed by health extension workers, female high school graduates originally from the sub-district and with one year of training in healthcare. Two health extension workers staff each health post. The nearest hospital is located in Butajira town, 30 km South of Buei town, the capital of the district. While this study was being conducted, no formal mental health care was being provided within the district although at present an integrated mental healthcare programme has started with the support of the PRIME project. The Sodo district was selected for PRIME because it is a relatively typical rural district for Ethiopia, and is located close to the research infra-structure of the Butajira research project on severe mental disorders and the Butajira Demographic Surveillance Site [23, 24]. The site is also within reasonable travel distance of specialist mental health services.

### Participants

Participants were consenting adults, aged 18 years and above, who had been residing in the district for at least six months. For the population-based study, participants were selected through simple random sampling of households from the total list of the district households followed by random sampling of one adult from each selected household. A total of 1485 participants were included in the study. This sample size was calculated to estimate changes in treatment coverage for common mental disorders (CMD) as a result of interventions of PRIME. The prevalence of CMD was assumed to be about 10 %, which is a conservative approximation of the prevalence of CMD in Ethiopian studies [25]. For the facility-based study, participants were selected by allocating numbers of participants for each health centre proportional to the number of attendees registered over a two week period. Following that, actual participants were recruited consecutively from the outpatient clinic over a period of one to two weeks until the target sample size was reached. The facility sample was part of a baseline study looking at the detection of depression and alcohol use disorder by primary care staff and was powered for this purpose [26]. No separate sample size calculation was undertaken for this study on suicidality. Both the community and facility samples come from the same geographical catchment areas and both surveys were conducted within the same six month period in 2014.

### Assessment of non-fatal suicidal behaviour and potential risk factors

The three categories of non-fatal suicidal behavior were suicidal ideation, suicidal plan and suicide attempt. The World Mental Health (WMH) Survey Initiative version of the World Health Organization (WHO) Composite International Diagnostic Interview (CIDI) [27] was adapted for assessing the 12 month prevalence of suicidal behaviour. A hierarchy of single item questions explored these behaviours: Suicidal ideation: “Have you thought of taking your life in the past 12 months?”; Suicide plan (asked of those who had suicidal ideation): “Did you ever make a plan for taking your own life at any time in the past 12 months?”; Suicide attempt (asked to those who answered they had ideation or a plan): “Have you attempted to take your own life in the past 12 months?” The help seeking items in the CIDI were also adapted to evaluate help seeking behaviour in the sample. The adaptation was primarily to include services relevant for the setting.

The CIDI was used previously in Ethiopia in various population-based studies [12, 13, 28] and had established reliability and acceptability [29].

The AUDIT [30] was developed by the World Health Organization (WHO) as a screening tool to indicate problematic consumption of alcohol in the previous 12 months in people attending primary care facilities [31]. The AUDIT has ten items, each rated on a four-point scale, giving a total score ranging from 0 to 40. Although not validated in the Ethiopian setting, the AUDIT has been used in neighbouring countries [32, 33]. Local alcoholic beverages were converted into standard equivalent alcohol units [34]. AUDIT score of 16 or more, corresponding with at least hazardous alcohol use, was considered a potential risk factor for suicidality.

Depressive symptoms were assessed using the 9-item patient health questionnaire (PHQ-9), a 9-item questionnaire developed to assess probable depression in the primary care setting [35]. The PHQ-9 could be used either as a continuous scale or the scores may be categorised into severity grades, typically as mild (score of 5-9), moderate (score of 10-14), moderately severe (score of 15-19) and severe (score of 20 and above). This latter categorisation can potentially assist clinical decision making, and we have used categories to establish severity of depression in modelling risk factors. The PHQ-9 has been validated in the general hospital setting in Ethiopia [36] as well as primary care settings in preparation for the current study [37].

Other potential risk factors included demographic characteristics, mainly gender, age and education status. Educational status was divided into three categories: illiterate, literate without formal education and formal education. These categories take into account the large number of people in Ethiopia who are able to read and write through various educational routes, such as religious programmes and the governmental literacy programmes.

### Administration of assessment instruments

Assessment instruments were administered in Amharic by lay data collectors trained for one week. The data collection was supervised in the field by trained degree-level supervisors. The instruments were piloted and pre-tested in selected sub-districts.

### Data management and analyses

Data were entered into Epi-data version 3.1 and analysed using the Statistical Packages for Social Sciences, version 20 (SPSS 20; IBM Corp 2012) and Stata version 13 (Stata for Windows). Descriptive analyses (i.e., means and percentages) were used to summarise the profile of the outcomes and relevant characteristics. Logistic regression models were fitted to assess the association of the main outcomes (suicidal ideation, suicidal plan and suicide attempt) with potential risk factors. Logistic regression model was also fitted to assess potential factors associated with transition from suicidal thoughts to suicide attempt. These potential risk factors were selected a priori based on evidence from existing literature and our theoretical assumption that these factors would be relevant for the outcomes of interest.

### Ethical considerations

The study was approved by the Scientific Committee of the Department of Psychiatry, Addis Ababa University, and the Institutional Review Board of the College of Health Sciences of Addis Ababa University. In all cases, informed consent was sought after adequate information about the study, and the potential benefits and risks, had been provided. Participants who were suicidal were referred for assessment by a psychiatric nurse or a psychiatrist employed or contracted by PRIME.

## Results

### Demographic characteristics

From the two settings, a total of 2499 participants, 1485 from the community and 1014 from health facilities, were recruited (Table 1). The non-response rate from potential participants approached in the community sample was 4.6 % (*n* = 71/1556) while there was no non-response in the facility sample. Over half of the participants were women (54.3 %) and married (71.0 %) and with some level of education (55.2 %). The vast majority of the participants were of the Gurage ethnic group (91.8 %) and Orthodox Christians (89.9 %). The livelihood of most participants depended on private sector work.

**Table 1:** Background characteristics of study participants

Characteristics	Overall		Community sample		Facility sample		
	Number	Percent	Number	Percent	Number	Percent	*p*-value**
Sex (*N* = 2499)							
Male	1142	45.7	679	45.7	463	45.7	0.975
Female	1357	54.3	806	54.3	551	54.3	
Age in years (*N* = 2493)							<0.001
<25	489	19.6	207	13.9	282	27.4	
25-34	710	28.5	424	28.6	286	28.4	
35-44	550	22.1	369	24.9	181	18.0	
45-54	311	12.5	205	13.8	106	10.5	
55+	433	17.4	280	18.9	153	15.2	
Marital status (*N* = 2499)							<0.001
Never married	447	17.9	178	12.0	269	26.5	
Currently married	1773	71.0	1134	76.4	639	63.0	
Widowed/divorced	279	11.2	173	11.6	106	10.5	
Religion (*N* = 2499)							<0.001
Orthodox	2247	89.9	1371	92.3	876	86.4	
Muslim	68	2.7	41	2.8	27	2.7	
Protestant	176	7.1	71	4.8	105	10.4	
Other	8	0.3	2	0.1	6	0.6	
Ethnicity (*N* = 2498)							0.001
Gurage	2292	91.8	1384	93.3	908	89.6	
Other	206	8.2	100	6.7	106	10.4	
Educational status (*N* = 2495)							<0.001
illiterate	1119	44.9	719	48.6	400	39.5	
Read and write	449	18.0	325	21.9	124	12.2	
Formal education	927 37.2		437	29.6	490	48.3	
Occupation (*N* = 2489)							<0.001
Housewife	595 23.9		337	22.8	258	25.5	
Private sector	1265	50.8	888	60.1	377	37.3	
Student	129	5.2	46	3.1	83	8.2	
Civil servant	160	6.4	35	2.4	125	12.4	
Other	340 13.7		171	11.6	169	16.7	

*In this setting, voluntary work is something people do until they find a job. **P values are assessing comparability of community and facility samples

### Prevalence non-fatal suicidal behaviour

The 12 month prevalence of any non-fatal suicidal behaviour was 7.9 % (*N* = 197/2498) (95 % CI = 6.8, 8.9); which was significantly higher in the facility (10.3 %; 95 % CI = 8.4, 12.1) than the community sample (6.3 %; 95 % CI = 5.0, 7.5) (Table 2). Nearly 6 % of participants expressed plans to commit suicide: 4.6 % of the community sample and 7.9 % of the facility sample, which was a statistically significant difference (*P* = 0.001). The percentage who reported attempting suicide was 3.8 % in the community sample and 5.4 % in the facility sample, with a combined prevalence of 4.4 % (95 % CI = 3.6, 5.3). Over half (56.4 %) of those with suicidal ideation, 60.2 % in the community sample and 52.9 % in the facility sample, had transitioned from ideation to attempt within the year.

**Table 2:** Non-fatal suicidal behaviour and service utilisation

Characteristics	Community sample		Facility sample	
	Number	Percent	Number	Percent
Thought of taking your life				
Yes	93	6.3	104	10.3
No	1391	93.7	910	89.7
Made a plan to take your own life				
Yes	68	73.1	80	76.9
No	25	27.2	24	23.1
Attempted to take your own life				
Yes	56	60.2	55	52.9
No	37	39.8	49	47.1
Discussed with any one about your thoughts or attempts of self harm				
Yes	21	22.6	57	54.8
No	72	77.4	47	45.2
With whom did you discuss?*				
Friend/neighbour	4/21	19.1	16/57	28.1
Spouse/partner	7/21	33.3	5/57	8.8
Other family member	9/21	42.9	13/57	22.8
Employer/co-worker	1/21	4.8	1/57	1.8
Traditional healer	0/21	0.0	----	----
Health care worker	1/21	4.8	0/57	0.0
Religious or spiritual advisor	----	---	1/57	1.8
Other people	----	---	21/57	36.8
Received any treatment for thinking about or attempting to take your own life				
Yes	10	10.8	9	8.7
No	83	88.2	95	91.3
PHQ total score for		Median (IQR)		Median (IQR)
Non- suicidal cases		2 (0-4)		3 (1-6)
Suicidal cases		8 (5-12)		9 (6-12)

*Multiple responses allowed. Because of this and rounding of decimals, percentages do not always add up to 100.0 %

### Help seeking

Out of 93 community respondents that reported suicidal thought or attempt in the last 12 months, 10.8 % (10/93) reported receiving some form of treatment (Table 2). Three of these reported receipt of treatment at a public health facility, mainly analgesics and related medical treatment, two counselling, although source of counselling was unclear, and three had traditional treatment (treatments based on local customs). None reported receiving psychothropic medications. The proportion in the facility sample who received care was slightly lower (8.7 %) but this was not statistically significant (*p* = 0.951). Overall, 60.4 % did not discuss their suicidal thoughts or acts with others and this was significantly higher in the community (77.4 %) than the facility sample (45.2 %) (*p* < 0.001). Those who had discussed with others mostly did so with their family members (n= 31/78;39.7 %).

### Factors associated with non-fatal suicidal behaviour and suicidal transition

Experience of depressive symptoms was the factor that was most strongly associated with non-fatal suicidal behaviour. With increasing severity of depressive symptomatology measured with the PHQ, the strength of association increased in all three categories of suicidal behaviour; suicidal ideation, plan and attempt (Table 3). Alcohol use disorder (AUDIT score of 16 and above) and Younger age (age <25 years) were also associated with all three forms of suicidal behaviour. The only factor associated with transition from suicidal ideation to suicidal attempt was scoring 10 and above on the PHQ (O.R = 2.73; 95 % CI: 1.19, 6.27).

**Table 3:** Association between background demographic and clinical characteristics and non-fatal suicidal behaviours in the last 12 months

Characteristics	Thought of committing suicide in the last 12 months (*n* = 2485)	Planned to commit suicide in the last 12 months (*n* = 2485)	Attempted suicide in the last 12 months (*n* = 2485)	Transition from thought to Attempt (*n* = 196)
	Adjusted Odds Ratio (aOR) (95 % Confidence Interval (CI))	aOR (95%CI)	aOR (95%CI)	aOR (95%CI)
Sex				
Male	Ref	Ref	Ref	Ref
Female	1.48 (0.97, 2.25)	1.58 (0.98, 2.56)	1.32 (0.76, 2.27)	0.96 (0.46, 2.06)
Age in years				
<25	2.73 (1.31, 5.69)	2.40(1.03, 5.55)	3.47 (1.33, 9.04)	1.83 (0.52, 6.48)
25-34	1.35 (0.78, 2.31)	1.46(0.80, 2.67)	1.71 (0.85, 3.45)	1.75 (0.63, 4.82)
35-44	1.48 (0.87, 2.52)	1.56 (0.86, 2.83)	1.85 (0.94, 3.66)	1.56 (0.58, 4.19)
45-54	1.14 (0.63, 2.05)	1.06(0.54, 2.08)	1.38 (0.65, 2.93)	1.36 (0.44, 4.20)
55+	Ref	Ref	Ref	Ref
Marital Status				
Never married	Ref	Ref	Ref	Ref
Currently married	1.55 (0.84, 2.85)	1.66(0.82, 3.36)	1.64 (0.74, 3.61)	1.06 (0.37, 3.00)
Formerly married	1.39 (0.62, 3.08)	1.30(0.52, 3.25)	1.50 (0.54, 4.23)	1.14 (0.29, 4.43)
Education				
Illiterate	Ref	Ref	Ref	Ref
Read and write	1.32 (0.83, 2.11)	1.47(0.87, 2.48)	1.15 (0.63, 2.08)	0.89 (0.38, 2.12)
Formal education	1.03 (0.65, 1.65)	1.07(0.63, 1.82)	0.89 (0.49, 1.62)	0.71 (0.30, 1.65)
PHQ9 total score				
<5	Ref	Ref	Ref	Ref
5-9	6.02 (3.97, 9.15)	7.03(4.24, 11.66)	7.33 (4.05, 13.39)	1.73 (0.75, 3.96)
10+	23.61 (15.19, 36.70)	27.39(16.29, 46.07)	30.78 (16.85, 56.24)	2.73 (1.19, 6.27)
Audit total score				
<16	Ref	Ref	Ref	Ref
16+	2.24 (1.21, 4.14)	2.77(1.43, 5.34)	2.72 (1.31, 5.68)	1.92(0.60, 6.17)
Sample type				
Facility	Ref	Ref	Ref	Ref
Community	0.82 (0.59, 1.15)	0.78 (0.53, 1.14)	1.02 (0.66, 1.58)	1.51 (0.80, 2.87)

## Discussion

Non-fatal suicidal behaviour is a relatively common problem in this predominantly rural community in Ethiopia. The current findings taken together with our previous observations in this population [25] of high levels of common mental disorders, alcohol use, and life events, are indications of the public health relevance of mental disorders.

Suicide attempt is one of the most crucial predictors of completed suicide, even four decades after the attempt had occurred, making attempted suicide an important target for the prevention of suicide [38]. Suicidal ideation is also in the pathway to completed suicide and would constitute an important target for prevention of suicide [39]. Although attending health services following suicide attempt or suicidal ideation offers an opportunity for prevention, in this study setting, most people with suicidal behaviours did not use health services. Communication of suicidality with family members, providers or other people was also very low. The low service uptake may be explained partly by the lack of accessible services; however, the low communication of suicidality with family and providers is an important barrier for the implementation of prevention strategies. Population level interventions should target the high levels of associated stigma and condemnation in the population [40] to encourage openness on the subject. Intervention programmes being developed and delivered by PRIME in Ethiopia [26], which include packages for enhancing awareness, community inclusiveness, empowering of providers and supporting health system change, have the potential to be beneficial, if tailored to suicidal behaviours.

Nevertheless, there are key gaps unaddressed in our study, which need to be considered in planning or providing interventions. This study did not explore the nature of the suicidality. For example, better information about the severity of the suicidality, the nature of the plans and what the means of the attempts were, would usefully inform public health intervention. In most rural developing country settings, organophosphate poisoning is a common method of suicide attempt and is associated with increased mortality due to lack of accessible health services [41]. Relevant triggers, such as social conflicts, family problems and financial strains should have been explored to identify targets for non-health interventions. Thus understanding the methods and immediate contexts of the attempt would inform the form and content of intervention to be provided for those with suicidal behaviour.

The factors associated with suicidality in this study, young age, alcohol use disorder and depressive symptoms, are consistent with what has been reported previously [16, 17]. However, the study was cross-sectional and thus causal interpretation of these associations can only be drawn based on previous studies.

The prevalence of non-fatal suicidal behaviour was relatively higher in this population compared to that reported in other populations in Ethiopia although rates are higher among those with symptoms of mental disorder. For example, whereas the lifetime prevalence of suicide attempt in the neighbouring district of Butajira was 3.2 % [8], the prevalence among those with minor depression in the neighbouring population of the Zay and Butajira was 5 and 14 % respectively [12, 13]. During a 10 year follow up of people with severe mental disorder, the rate of suicide attempt varied from 13 % in schizophrenia to 26 % in major depressive disorder [11].

Reason for the comparatively higher rate of suicidality in this study population may relate to the relatively higher level of mental distress, alcohol use and life events against a lower level of social support shown in our previous report [25]. This finding is also consistent with the relatively high rate of persistent suicidal preoccupations and suicide attempt assessed over a 30 day period in the same district [25]. Therefore, this higher rate of suicidality may be partly a reflection of the overall level of unmet mental health need in this population. A more detailed exploration of the context and factors contributing to suicide would have been relevant in determining the content of intervention and this is an important limitation of our study. Follow-up of this sample would also help determine shorter term clinical implications of these findings and should be considered. Although the cross-sectional nature of the study has the known limitations of recall bias and makes attribution of risk factors difficult, the study is adequate for establishing the magnitude of the problem of suicidality. Combining the community and facility sample may also introduce bias. However, the two samples were drawn from the same population (the Sodo district population) and combining would give us more power to detect relevant risk factors, especially in the analysis of suicidal attempt, which is a relatively rare but of higher public health importance. We present data separately before combining, which would allow interpretation of data separately. We also adjust for the source of the participant population (facility vs community) while investigating the role of other risk factors with larger sample size. Despite significant differences in the background characteristics of the two samples, study group was not a significant predictor of the target outcomes in the multivariable model. Finally, given this is a cross-sectional survey, the concept of transition from ideation to attempt may be considered inappropriate. Theoretically the two could occur at the same time although reverse causality is virtually impossible. Nevertheless, what has been attempted here is more pragmatic and the assumption has been made that, even when an attempt happens without premeditation, there is a degree of thinking that precedes any attempt.

## Conclusion

The recent emphasis of the World Health Assembly is an important milestone in the context of findings such as ours suggesting a higher burden of disease related to suicidal behaviour. The WHO mental health action plan 2013-2020, identifies broad factors relevant for suicide and proposes multi-sectoral approach for the prevention of suicide. Furthermore, the action plan commits governments to reducing suicide by 10 %. Our data, complementing the sparse data originating from low and middle income countries, reveals that the hidden burden of suicidal behaviour is substantial. In the broader context of stigma and condemnation that may keep these behaviours hidden, public interventions to improve stigma are important. This study also indicates that addressing the need of young people, improving access to care for depression and alcohol use disorder are important next steps. Furthermore, we recommend further studies to understand the nature of the behaviour and context in which the behaviour develops, as well as developing effective brief interventions for the behaviour.

## Ethics approval and consent to participate

The study was approved by the Institutional Ethics Review Board of the College of Health Sciences of Addis Ababa University ***(REF. 084/11/PSY)***. All participants took part in the study after providing informed consent.

## Consent for publication

N/A.

## Availability of data and materials

The data is part of an ongoing cross-country study, Programme for Improving Mental Healthcare (PRIME). Because of the ongoing and cross-country nature of the study, we are not able to make the data publicly available at present. However, data will be made available once the study is completed in the next 3 years.

## Abbreviations

AUDIT: Alcohol Use Disorder Investigation Test
CIDI: Composite International Diagnostic Interview
CMD: Common Mental Disorders
LMICs: Low and Middle Income Countries
PHQ: Patient Health Questionnaire
PRIME: Programme for Improving Mental Healthcare
SNNPR: Southern Nations, Nationalities and Peoples Region
SPSS: Statistical Packages for Social Sciences
WHO: World Health Organization
WMH: World Mental Health
